# An Overview of Clinical Outcomes in Transvenous and Subcutaneous ICD Patients

**DOI:** 10.1007/s11886-018-1021-8

**Published:** 2018-07-10

**Authors:** S. W. E. Baalman, A. B. E. Quast, T. F. Brouwer, R. E. Knops

**Affiliations:** Heart Center, Department of Clinical and Experimental Cardiology, Amsterdam Medical Center, University of Amsterdam, PO Box 22700, 1100 Amsterdam, DE The Netherlands

**Keywords:** Subcutaneous implantable cardioverter defibrillator, Transvenous implantable cardioverter defibrillator, Appropriate shocks, Inappropriate shocks, Infections, Complications

## Abstract

**Purpose of Review:**

Clear guidelines on when to select a subcutaneous ICD (S-ICD) over a transvenous ICD (TV-ICD) are lacking. This review will provide an overview of the most recent clinical data on S-ICD and TV-ICD therapy by pooling comparison studies in order to aid clinical decision making.

**Recent Findings:**

Pooling of observational-matched studies demonstrated an incidence rate ratio (IRR) for device-related complication of 0.90 (95% CI 0.58–1.42) and IRR for lead-related complications of 0.15 (95% CI 0.06–0.39) in favor of S-ICD. The IRR for device infections was 2.00 (95% CI 0.95–4.22) in favor of TV-ICD. Both appropriate shocks (IRR 0.67 (95% CI 0.42–1.06)) and inappropriate shocks (IRR 1.17 (95% CI 0.77–1.79)) did not differ significantly between both groups.

**Summary:**

With randomized data underway, the observational data demonstrate that the S-ICD is associated with reduced lead complications, but this has not yet resulted in a significant reduction in total number of complications compared to TV-ICDs. New technologies are expected to make the S-ICD a more attractive alternative.

## Introduction

Since the first implantable cardioverter defibrillator (ICD) was introduced in 1980, ICDs provide lifesaving therapy for patients at risk for ventricular arrhythmias [1, 2]. Transvenous ICD (TV-ICD) systems have been the first-line therapy since their introduction in the early 1990s. However, besides their lifesaving capacities, the transvenous leads carry their own risk. Lead-related complications and systemic infections are severe side effects causing significant morbidity and mortality [3].

The subcutaneous ICD (S-ICD) was designed to reduce lead-related complications and reducing the risks associated with systemic infections and device extractions, by creating an extra-thoracic, implantable, defibrillator system without the need for venous access [4]. Consisting a single lead placed on the sternum, the S-ICD is not able to provide chronic pacing except from 30 s on demand post-shock pacing. In the absence of chronic pacing capabilities, the S-ICD is not suitable for patients requiring either bradycardia, anti-tachycardia (ATP), or cardiac resynchronization pacing.

In the past years, several studies have shown similar safety and efficacy of the S-ICD [5, 6]. A recent meta-analysis compared observational data of outcomes in patients implanted with TV-ICD and S-ICDs [7], but currently no randomized data is available. The ongoing trial, a Prospective, Randomized Comparison of Subcutaneous and Transvenous Implantable Cardioverter Defibrillator Therapy (PRAETORIAN) trial, will be the first randomized study comparing both devices head-to-head (ClinicalTrials.gov NCT01296022), of which the first results are expected in 2020 [8]. In addition, the randomized trial Avoid Transvenous Leads in Appropriate Subjects (ATLAS S-ICD), which started enrollment last year, will also compare single-chamber TV-ICDs with S-ICDs (ClincialTrials.gov NCT02881255). In the absence of randomized data, the latest ESC guidelines for the management of patients with ventricular arrhythmias and the prevention of sudden cardiac death now give the S-ICD a class IIa recommendation for patients without need of bradycardia, anti-tachycardia pacing (ATP), or cardiac resynchronization therapy [9]. The recently published AHA/ACC/HRS guideline for management of patients with ventricular arrhythmias and the prevention of sudden cardiac death give a S-ICD class I recommendation for patients at high risk for infection or without adequate venous access, without an indication for pacing or ATP [10]. As clear guidelines on when to select a subcutaneous ICD (S-ICD) over a transvenous ICD (TV-ICD) are lacking, the aim of this review is to provide a pooled overview of the clinical outcomes and discuss device selection considerations in order to aid physicians in selecting the optimal device for the individual patient.

## Methods

A previously published systematic review and meta-analysis included five matched head-to-head comparison studies [7]. Since the publication of this meta-analysis, several new matched comparisons have been published including one with more patient years (PYs) of follow-up than the previous meta-analysis [11–13]. Therefore, in this review, we added the most recently published studies to the existing meta-analysis and pooled the results for clinical outcomes. The outcomes were device-related complications, lead complications, device infection, appropriate shocks (AS) and inappropriate shocks (IAS). We present an incidence rate ratio (IRR) as this methodology corrects the number of events for the duration patients have been followed as studies had widely ranging follow-up durations. We obtained the IRR from the random effects model. Of the included studies, two of the seven only evaluated short-term in hospital outcomes [13, 14]. The heterogeneity, variation in study outcomes between studies, is assessed by using *I*^2^ statistic, with *I*^2^ > 50% indicating significant heterogeneity.

## Results

**Fig. 1:**
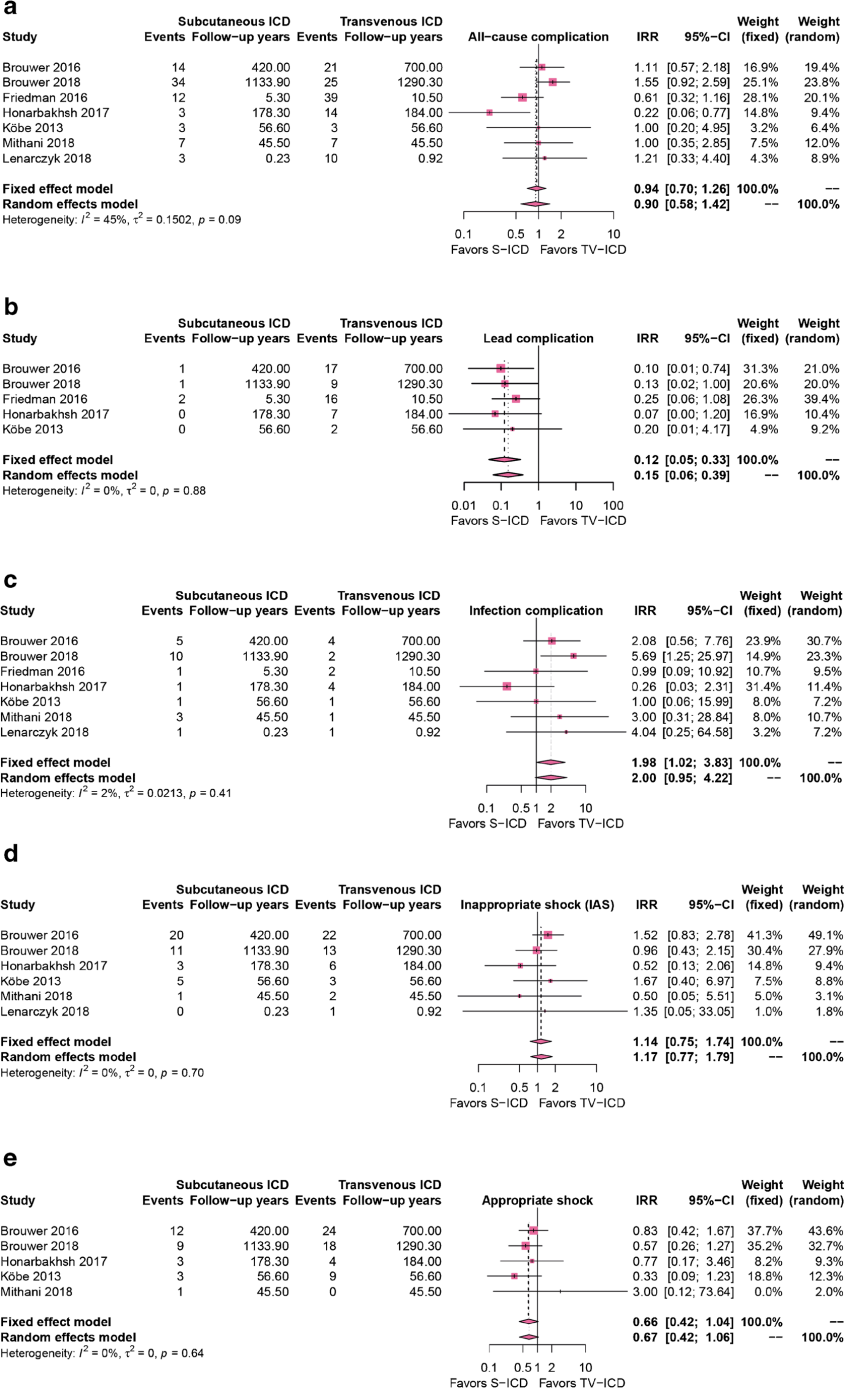
**a** All-cause complications in the S-ICD group compared to the TV-ICD group. There is no difference between both groups. **b** Lead-related complications in the S-ICD group compared to the TV-ICD group. Fewer lead-related complications occurred in the S-ICD group. **c** Infection-related complications in the S-ICD group compared to the TV-ICD group. There is no significant difference between both groups. d + e inappropriate shocks (IAS) and appropriate shocks (AS) in the S-ICD group compared to the TV-ICD group. There is no significant difference in AS or IAS between both groups. IRR = incidence rate ratio; CI = confidence interval; S-ICD = subcutaneous implantable cardioverter defibrillator; TV-ICD = transvenous implantable cardioverter defibrillator.

Seven studies were included in this analysis [11–17]. The total number of included PYs was 1840 PYs in the S-ICD group versus 2288 PYs in the TV-ICD group. Figure 1a shows the device-related complication rate of all seven studies comparing TV-ICDs with S-ICDs. The pooled IRR of all-cause complications was 0.90 (95% CI 0.58–1.42), non-significantly in favor of the S-ICD. Five of the seven studies reported lead-related complications (Fig. 1b) and all five showed a decrease in favor of the S-ICD. The pooled IRR was 0.15 (95% CI 0.06–0.33), in favor of the S-ICD with 0% heterogeneity between studies. All seven studies compared infections in TV-ICDs versus S-ICDs (Fig. 1c). The pooled IRR was 2.00 (95% CI 0.95–4.22), suggesting more infection-related complications in S-ICD patients.

IAS were reported by six of the seven studies (Fig. 1d). The pooled IRR was 1.17 (95% CI 0.77–1.79), non-significantly in favor of TV-ICDs. All studies reporting the cause of IAS showed that those in TV-ICD, patients were primarily for supraventricular tachycardia and those in S-ICD due to T-wave oversensing (TWOS). Five out of the seven studies reported AS and four showed an increase in AS in TV-ICD patients [11, 12, 15–17]. The pooled IRR was 0.68 (95% CI 0.42–1.03), non-significantly in favor of S-ICDs (Fig. 1e).

## Discussion

### Pooled Clinical Outcomes

Pooling of studies reporting clinical outcomes with the first-generation S-ICD in 1840 patient years follow-up demonstrates that lead-related complications are significantly reduced, but this does not translate into a reduction of all-cause complications. There is a trend towards more device infections in S-ICD patients. Both AS and IAS do not differ significantly, although there is a trend towards fewer AS and more IAS in S-ICD.

### Lead-Related Complications

In the National Cardiovascular Data Registry (NCDR), lead dislodgement was the most common complication seen in primo TV-ICD patients [18]. Kleeman et al. reported an increasing annual lead failure rate reaching 20% in leads of 10 years old [19]. The S-ICD effectively eliminates these lead complications. Besides lead failure or dislodgement, damage to the tricuspid valve apparatus caused by transvenous leads has been described and this is avoided in S-ICD recipients [20]. Valve damage may result in right-sided heart failure, particularly in older and sicker patients such as those with a low ejection fraction, who make up the majority of the ICD population. Future studies are needed to analyze the value of S-ICD implantation over TV-ICD on tricuspid valve damage in patients implanted with an ICD. This may lead to a further reduction in lead-related complications in S-ICD patients compared to TV-ICD patients.

### Infections

Our pooled study showed a trend towards more device infections in S-ICD patients, with device infections defined as both local and systemic infections. All but one S-ICD infections were local infections. The only systemic infection occurred in a S-ICD patient with a concomitant transvenous pacemaker in situ [15]. Although lead endocarditis is a rare complication, it is associated with significant morbidity and mortality of up to 30% at within 1 year [21].

### Appropriate and Inappropriate Shocks

There is a trend towards fewer AS in S-ICD patients. This is likely caused by a longer programmed time to therapy and high-rate cutoffs zones in S-ICD, which may allow spontaneous termination of VTs. The ongoing randomized PRAETORIAN trial with pre-specified device programming in both arms will determine whether there is a true difference in appropriate shock rate.

The IAS rate in S-ICD studies is often higher than what has been reported in the TV-ICD therapy reduction programming trials. The difference is at least in part caused by patient characteristics such as age and the absence of heart failure, as this pooled analysis of matched patients 1840 PY in the S-ICD group versus 2288 PY in the TV-ICD group failed to find a significant difference in the IAS rate. The nature of the IAS differed between devices: in TV-ICD patients, supraventricular arrhythmias are the main cause, whereas TWOS was the main cause in S-ICD patients [6, 22, 23]. A new S-ICD sensing methodology that incorporates a high-pass filter (SMARTPASS) has been demonstrated to reduce the number of patients with inappropriate shocks by 50% and the inappropriate shock burden by 68% [24•]. In patients with the SMARTPASS algorithm, the inappropriate shock rate is now in the same range as in patients implanted with single-chamber TV-ICDs at 1-year follow-up [24•].

## Considerations for Patient Selection

### Bradycardia and Anti-tachycardia Pacing

In the absence of chronic pacing capabilities in S-ICDs, it is important to determine whether there is a need for pacing. In the single-chamber ICD population, only a minority of ICD patients actually require pacing or develop this need for pacing during follow-up [25]. Bradycardia pacing should be reserved for patients with a class I pacemaker indication such as patients with sinus node dysfunction or high-grade AV block who present with symptomatic bradycardia of chronotropic incompetence [26, 27]. While ATP provides the ability to terminate ventricular tachycardia without a shock, patients actually receiving ATP with longer therapy delays and higher cutoff rates were 4 and 8% respectively in the MADIT-RIT trial [28]. However, it is important to emphasize that ICD patients presenting with monomorphic ventricular tachycardia are best treated by medication, revascularization, or ablation. ATP is a modality to terminate ventricular arrhythmias but it does not treat the underlying disease or cause of arrhythmias. In the near future, an ATP-enabled leadless cardiac pacemaker (LCP) is expected and will make ATP delivery possible on demand by the S-ICD using conductive device-device communication between the S-ICD and LCP [29••].

### Anesthesia

In contrast to TV-ICDs, approximately half of all S-ICD implants are performed under general anesthesia (GA), as it requires creation of a larger device pocket in a more innervated area. GA is not available in every center around the clock for device implants, is associated with higher costs, and may lead to hemodynamic derailment, especially in frail patients with cardiac comorbidities, such as low ejection fraction or aortic valve stenosis. Recently, Miller et al. and Droghetti et al. showed the use of regional truncal blocks, such as the serratus anterior and transverse thoracic plane block, can be used in combination with monitored anesthesia care (MAC) to achieve excellent pain control [30–32]. These developments may in time eliminate the need for GA.

### Defibrillation Testing

The current guidelines recommend routine defibrillation or conversion testing (DFT) after every S-ICD implant [33]. The DFT is considered to be the ultimate test of optimal system positioning. Data from the EFFORTLESS and IDE studies have shown a high conversion rate during DFT for the S-ICD and that the first shock efficacy is similar to that of TV-ICDs [6], indicating that omitting DFT in S-ICD patients may be reasonable. However, during S-ICD implants, fluoroscopy is often not used and R-wave amplitude on the subcutaneous electrogram does not confirm adequate positioning. A computer modeling study has demonstrated that implant position and the presence of fat tissue under the can or coil of the device have both a large impact on the defibrillation threshold [34•]. Until evidence shows that S-ICD implantation without DFT is safe, it is recommended to routinely test every S-ICD implant without a contraindication. The recently started A Prospective Randomised Comparative trial of Subcutaneous Implantable Cardioverter-Defibrillator Implantation with and without Defibrillation Testing (PRAETORIAN-DFT) trial will randomize patients to a strategy in which patients are routinely tested versus not tested (ClinicalTrials.gov NCT03495297). In the non-testing arm of the study, the post-procedural chest x-ray will be used to assess S-ICD system positioning and generator replacement.

## Conclusions

The S-ICD has proven to be a good alternative for TV-ICD implantation. With randomized data underway, the observational data demonstrates that the S-ICD is associated with reduced lead complications, but this has not yet resulted in a significant reduction in the total number of complication compared to TV-ICDs. Since the introduction of the S-ICD, important steps have been made to facilitate widespread adoption. New technologies such as a communicating leadless pacemaker with ATP capabilities and SMARTPASS for IAS reduction will make the S-ICD an even more attractive alternative, and new strategies for both anesthesia and defibrillation testing may soon reduce the logistical complexity of S-ICD implantation.
